# Shear Capacity of Reinforced Concrete Beams under Monotonic and Cyclic Loads: Experiments and Computational Models

**DOI:** 10.3390/ma14154092

**Published:** 2021-07-22

**Authors:** Kamil Bacharz, Barbara Goszczyńska

**Affiliations:** Faculty of Civil Engineering and Architecture, Kielce University of Technology, Al. Tysiąclecia Państwa Polskiego 7, 25-314 Kielce, Poland; bgoszczynska@tu.kielce.pl

**Keywords:** reinforced concrete beams, support zone, shear load capacity, calculation models, shear reinforcement, cyclic load

## Abstract

The paper reports the results of a comparative analysis of the experimental shear capacity obtained from the tests of reinforced concrete beams with various static schemes, loading modes and programs, and the shear capacity calculated using selected models. Single-span and two-span reinforced concrete beams under monotonic and cyclic loads were considered in the analysis. The computational models were selected based on their application to engineering practice, i.e., the approaches implemented in the European and US provisions. Due to the changing strength characteristics of concrete, the analysis was also focused on concrete contribution in the shear capacity of reinforced concrete beams in the cracked phase and on the angle of inclination of diagonal struts. During the laboratory tests, a modern ARAMIS digital image correlation (DIC) system was used for tracking the formation and development of diagonal cracks.

## 1. Introduction

The issue of the load-bearing capacity of reinforced concrete (RC) beams in the support zone, though extensively studied for years, still lacks proper understanding [1]. The problem is complex. Due to a complex state of stresses in the support zone, the primary stress trajectory is not parallel to the axis of the element. The problem becomes even more complex when reinforced concrete is used as a construction material. Reinforced concrete is a conglomerate composed of two interacting materials, concrete and steel, with different properties and the concrete matrix consists of various size aggregate particles randomly distributed in the cement paste. High heterogeneity of the material causes its parameters to rely on multiple factors. At service loads, reinforced concrete members go through two different stages, uncracked and cracked, which alter the state of internal stresses, leading to a change in concrete behavior. All this renders the description of RC member behavior under load a very challenging task and requires the adoption of many simplifying assumptions that must be verified experimentally. Considering that concrete and steel with increased strengths are more commonly used today (high and ultra-high-strength concrete and high-strength and high-ductility steel), the behavior description, particularly in the support zones, is invariably relevant. Determining maximum values of major tensile stresses occurring in the support zone can be limited, with some simplifications, to determining shear stresses in the neutral axis. For this reason, the bearing capacity is most often referred to as shear capacity.

Solving the shear problem theoretically seems virtually impossible, hence the need for calculation models that can describe, as precisely as possible, the behavior of the support zones of RC beams under load. Theoretical and experimental studies show (e.g., the works of R. Walther [2]) that shear capacity depends both on the shear force and the bending moment. The separation of these impacts greatly simplifies the design but fails to fully reflect the actual bearing capacity of the RC beam support zone. The topic remains open and needs to be pursued despite a long-term research effort for developing a general computational model able to faithfully describe the support zone behavior in RC beams under load. This need is evidenced by continuous changes introduced or planned to be introduced in standard recommendations, for example, in fib Model Code 2010 (MC2010) [3] or the US provisions, ACI 318-19 [4].

Advances in the use of RC structures, bold realizations of engineering and monumental structures, and their mass scale push engineers and researchers to find savings in the consumption of concrete and steel. As a result, concrete and steel technology is improving [5,6,7]. Targeted additives and admixtures [8], higher strength properties of steel and concrete create the need to update the approach to safe dimensioning of RC elements based on experimentally verified theoretical assumptions [9].

This work aims to present a comparative analysis of the shear capacity of RC beams with various static schemes and loading modes and programs obtained experimentally and computationally from selected models. It is important to note here that both the static scheme and the beam reinforcement were designed to ensure shear failure. The results obtained were the basis for determining the experimental shear capacity of the beams. Comparative analysis was based on numerical models chosen to fit different theoretical assumptions.

The analysis uses test results for single-span and double-span reinforced concrete beams loaded to failure in a monotonic and cyclic manner. The computational models were selected in the context of their application to engineering practice, including the approaches implemented in the European and US provisions. Due to the changing strength characteristics of structural concrete, the analysis was designed to include the concrete contribution in the shear capacity of cracked RC beams. The adopted testing program for single span simply supported beams and those with two spans under monotonic and variable loads was assumed to allow establishing the element that would be a high-credibility numerical model verification pattern. During the laboratory loading tests, a modern ARAMIS digital image correlation (DIC) system was used to allow continuous tracking of displacements and crack (incl. diagonal cracks) formation and development.

## 2. Materials and Methods

### 2.1. Test Elements

**Figure 1:**
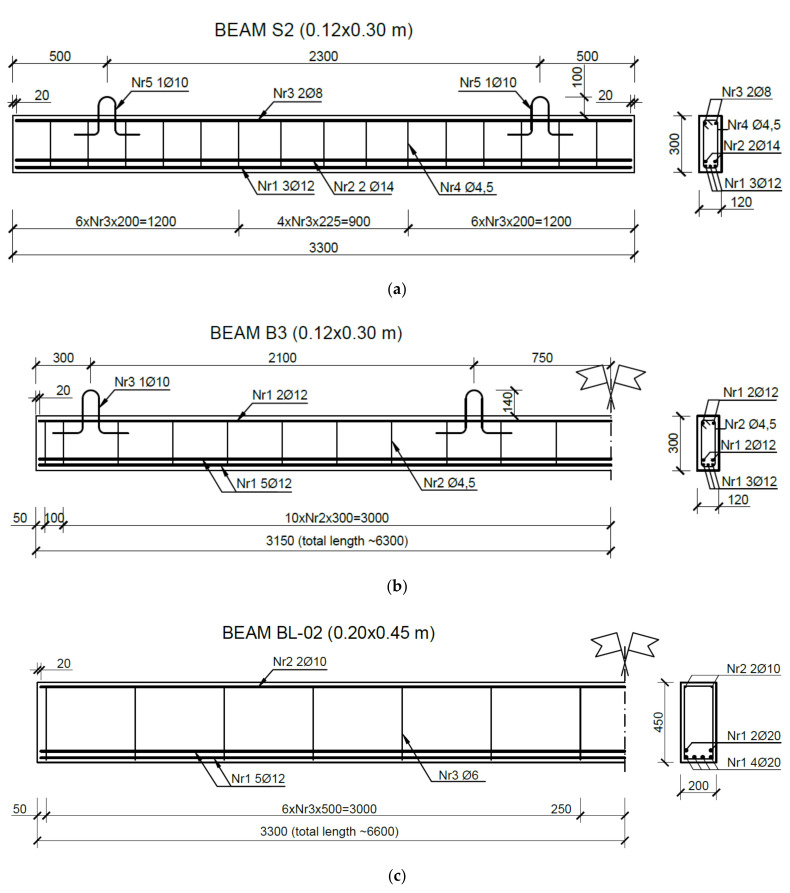
Reinforcement in beams (**a**) S2, (**b**) B3, (**c**) BL-02.

The tests were conducted on 11 RC beams loaded to shear failure. The beams were made from concrete in a prefabrication plant to the designed class C40/50 and steel B500SP with a characteristic yield strength f_yk_ = 500 MPa and class C. The designed concrete class was used for the production of reinforced concrete bridge members. The composition of the developed mixture was provided by an automatic concrete batching plant. The tested beams differed in length, cross-sectional dimensions, reinforcement structure, loading program and static scheme. The list of elements with the adopted symbols is presented in Table 1. The letter M in the beam symbol indicates monotonic loading increasing to failure, and the letter C—low-cycle loading. The structure of the reinforcement is shown in Figure 1.

The cross-sections of the longitudinal and shear reinforcement used in the tested elements, along with the calculated reinforcement ratio, are summarized in Table 2. To obtain shear failure, the reinforcement system had to provide significant bending capacity at relatively low shear capacity. For this purpose, a relatively high ratio of longitudinal reinforcement (0.02) was assumed (bars in the tension zone), and the spacing of stirrups approximately met the minimum shear reinforcement ratio, which according to Eurocode 2 is 0.0011.

**Figure 2:**
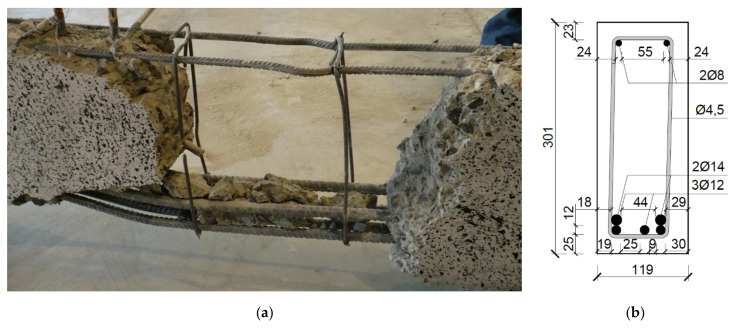
Survey of reinforcement distribution in the tested beams (**a**) view of the inside of the beam, and (**b**) the beam cross section with the actual location of the bars.

The average reinforcement ratio given in Table 2 results from the actual dimensions of the beam sections and the actual arrangement of the bars, which were obtained from the structural survey, illustrated, for example, in Figure 2. The survey was performed after loading the beam to failure and crushing it outside of the failure zone. Measurements were performed to determine the actual location of the centers of gravity of the tension reinforcement d_1_ and compression reinforcement d_2_, the effective depth of the section d and the lever arm of internal forces z.

In the subsequent analyses, for a given type of beam reinforcement structure, averaged results of the reinforcement ratio were used. Due to different stirrup spacing on individual sections of the beam, the shear reinforcement ratio (Table 2) was determined for the support zone (ρ_w_).

### 2.2. Test Setups

**Figure 3:**
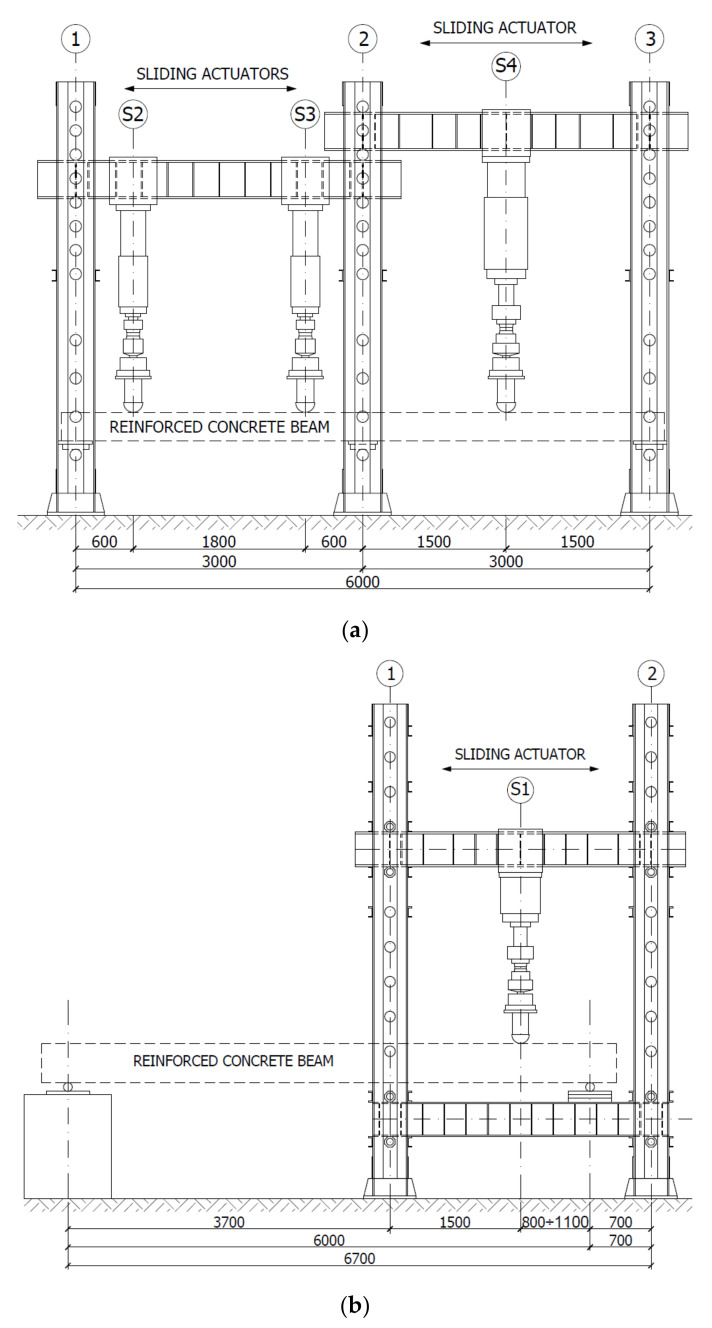
Schematic view of the test setups for beams (**a**) S2M, S2C, B3C, (**b**) BL-02M.

Figure 3 shows the test setups attached to the testing facility for single-span and double-span beams with span lengths of 3.00 m and 6.00 m, measured in the axes of the supports. The load programs were feasible as the test facility was equipped with a power supply system and a possibility of programming the load provided by the controller and a set of actuators. The load was applied using hydraulic actuators denoted as S1—1000 kN, S2 and S3—400 kN, and S4—600 kN.

**Figure 4:**
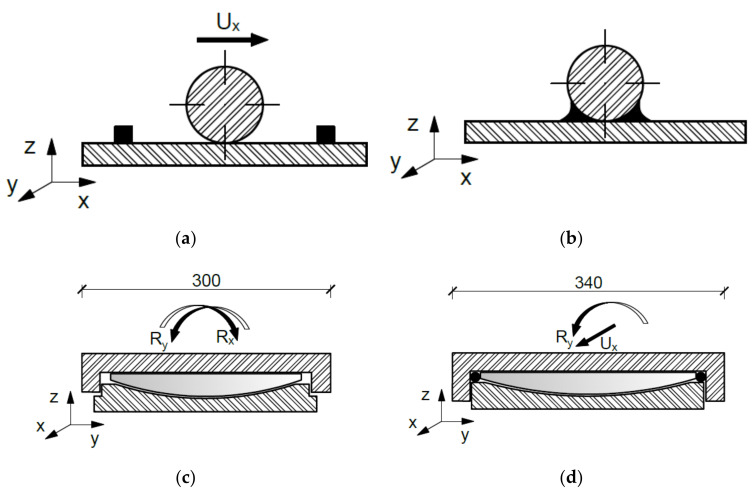
Types of used supports: (**a**) sliding point support, (**b**) non-sliding point support, (**c**) wide (bearing) sliding support, (**d**) wide non-sliding support.

In line with the adopted static test scheme, two types of supports, point and wide, were used, as shown in Figure 4. Point supports were made in the form of steel cylinders welded (non-sliding support) or not (sliding support) to a flat bar (beams BL-02M). The support called the wide support was made in the form of sliding and non-sliding bridge spherical bearings (beams S2M, C and B3).

### 2.3. Static Scheme and Load Program

**Figure 5:**
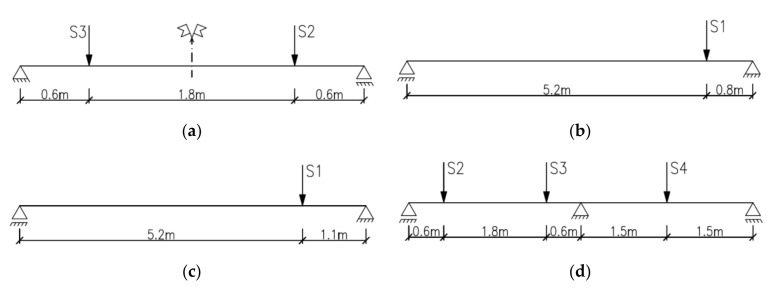
Static schemes (**a**) beams S2M and S2C, (**b**) beam BL-02M-1 (**c**) beams BL-02M-2 and 3 (**d**) beams B3C.

**Figure 6:**
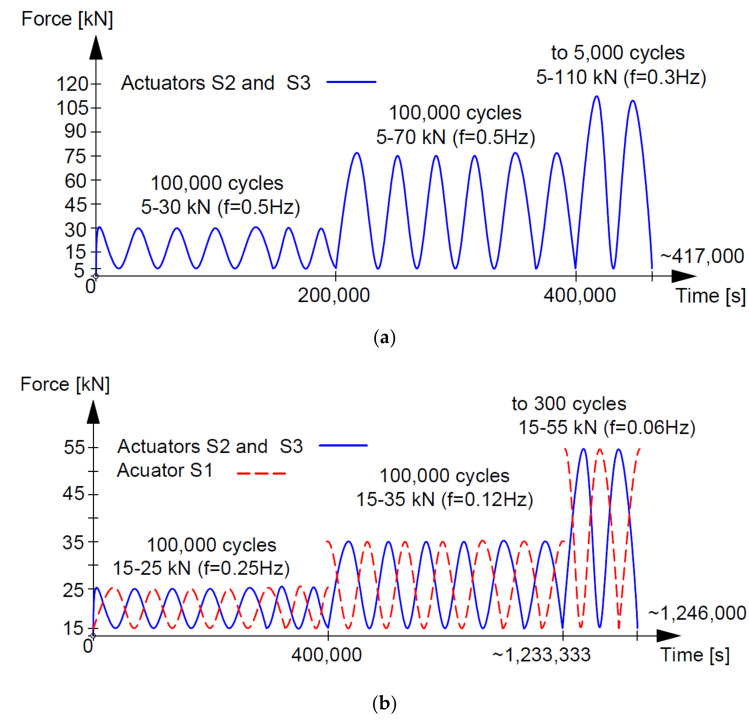
Low-cycle load program for beams (**a**) S2C (**b**) B3C.

Two static schemes were implemented for testing, as shown in Figure 5. Beams denoted S2M-1 and 2, S2C-1 and 2 (Figure 5a), BL-02M-1, 3 and 4 (Figure 5b,c) were tested as statically determinate single-span beams with a span in the support axes of 3.00 m and 6.00 m, respectively. Beams denoted as B3C-1 and 2 were statically indeterminate two-span elements (Figure 5d) with an equal span length of 3.00 m. For shear failure, loads in the form of concentrated forces were applied close to the supports, thereby achieving a relatively strong influence of the transverse force on capacity against a small influence of the bending moment. The S2M and S2C beams were loaded using two actuators (S2 and S3) placed at a distance of 0.60 m from the axis of the supports (Figure 5a). The S2M beams were loaded to failure with two equal monotonically increasing forces, while the loading of the S2C beams was low-cyclic (Figure 6a). The beam denoted as BL-02M-1 was loaded monotonically to failure with one concentrated force (S1) applied at a distance of 0.8 m from the support axis (Figure 5b), and the beam BL-02M-3 and 4 at a distance of 1.1 m from the support axis (Figure 5c). The monotonic load was characterized by a constant increase in force at a rate of 0.40 kN/min until the elements were destroyed.

The two-span beam static scheme was adopted for two elements denoted as B3C-1 and 2. The load consisted of three concentrated forces applied from three actuators, S2, S3 and S4, arranged along the length of the beam as shown in Figure 5d. Variable low-cyclic loads were applied to S2C-1 and 2 and B3C-1 and 2 beams in three ranges 100,000 cycles each, while for technical reasons, the force value did not go to zero. The upper values of the forces in the individual load ranges were assumed successively at levels corresponding to approximately 0.30; 0.50; and 0.70 of the expected destructive load. Due to the adopted ranges of forces and hardware capabilities, the load frequencies f ranged from 1 Hz to 0.06 Hz. A list of load ranges for individual beams, along with the frequencies used, is given in Table 3.

Beams S2C-1 and 2 were loaded with two forces acting simultaneously within the given ranges. Beams B3C-1 and 2 were loaded with three forces (Figure 5d); two on one span (S2 and S3) and one on the other span (S4). The load on the spans changed sinusoidally with a phase shift of 180⁰. The load on individual spans was alternating; during the increase in the load on one span, actuators S2 and S3, the other span was unloaded, actuator S4. Then the situation reversed and during the increase in the load on the span provided by the S4 actuator, the span loaded with the S2 and S3 actuators was unloaded. The described loading program is shown in Figure 6b, where the continuous blue line indicates loading with two forces, and the dashed red line indicates loading with one force. The cyclic loading programs indicate that although all ranges were intended to be 100,000 cycles, the last load range was reduced to 300 cycles due to the failure of the beams.

### 2.4. Test Apparatus

**Figure 7:**
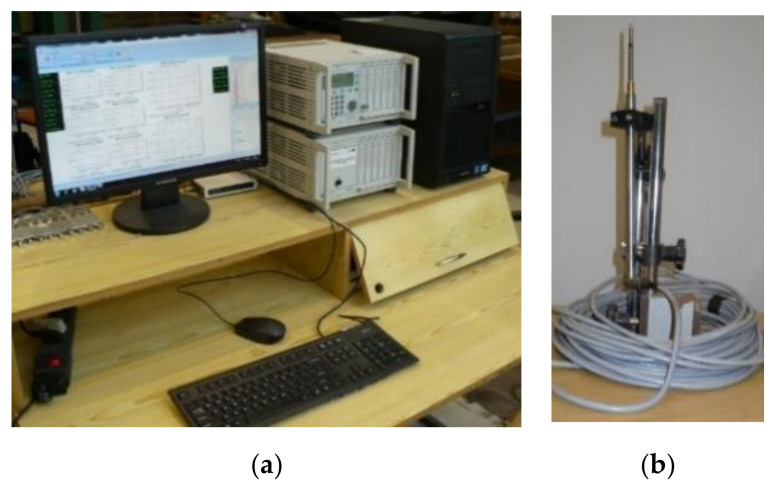
HBM measuring set (**a**) central unit, (**b**) distance sensor.

**Figure 8:**
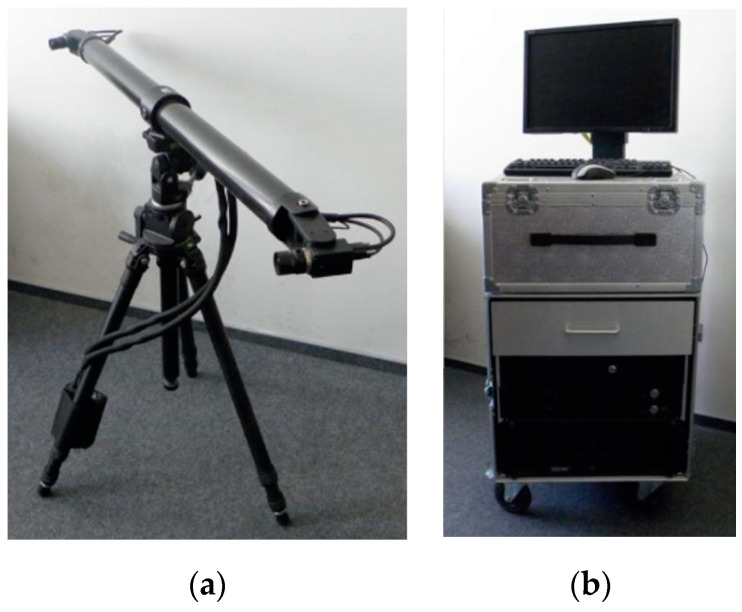
The optical measuring system (**a**) tripod with cameras, (**b**) central unit.

In addition to actuators enabling the implementation of the loading program, measuring equipment was used, which included the HBM measuring set and the DIC system (ARAMIS) [10,11]. The Hottinger Baldwin Messtechnik measurement set, consisting of two modular measuring amplifiers and inductive transducers (road sensors), recorded loads and displacements (Figure 7). The optical measuring system, ARAMIS, [10,11], was applied to analyze, calculate and document strain fields for the prepared beam surfaces (Figure 8). Additionally, using the analogue voltage outputs of the load controller, the entire test apparatus was synchronized, and the automatically set force was used to control the measurements performed by the HBM and optical systems [12]. This synchronization enabled assigning deflection results and the strain field image to each force value, which was also used to control the correctness of the measurements.

Five displacement sensors with a measuring range of 0.05 m were used for testing the single-span beams and ten sensors for testing the two-span beams. The sensors were placed at the beam midspan, under the force application points, and at a distance of ~0.25 m from the axis of the supports. Beam deflections measured with the HBM measuring set were used to verify the deformation results obtained from the optical measuring system. The deflections read from the sensors to an accuracy of 0.01 mm had the same values. 

The DIC system (ARAMIS) is used for non-contact, three-dimensional measurements of the surface deformation located within the measurement field range. It performs analyses and calculations and documents strains, allowing a graphical representation of results in a color scale. The calculations use a series of photos from two digital cameras. The strain of the pattern put on the side surface of the tested element is identified. The first photo in the series is taken as a photo of the component before loading and serves as the point of reference. After taking all the photos, the program compares them by assigning small square or rectangular planes, called facets, to the tested surface, and finds these facets in subsequent photos. Owing to the preparation of the tested surface, each facet has one and unique structure of black points. The system program compares the displacements of characteristic points (centers of facets). The device uses two-image photogrammetry for measurements [13]. This allows non-contact measurements that do not interfere with the loading process. The DIC system (ARAMIS) [11,12] (Figure 8) used in this study was equipped with two tripods with two cameras each. The system made it possible to measure strains, spatial deformations and cracks on the side surface of the beams during the entire loading process on two surfaces measuring 2.00 m × 1.50 m.

**Figure 9:**
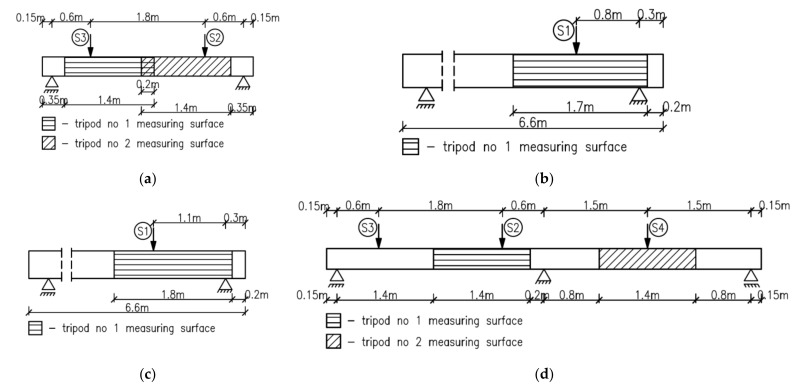
Measuring surfaces of the optical system for beams (**a**) S2M-1, 2 and S2C-1, 2, (**b**) BL-02-M-1, (**c**) BL-02-M-3, 4, (**d**) B3C-1, 2 (double-span beams).

Considering the dimensions of the tested elements and the force distribution, the following measurement fields were adopted:

- for elements S2M and S2C, two tripods of the optical system allowed testing two beam support surfaces by creating overlapping measurement fields with dimensions 0.30 m × 1.40 m (Figure 9a),
- for BL-02M elements, due to loading with one concentrated force, the measurement field covered the support zone at the actuator with dimensions of 0.45 m × 1.70 m and 0.45 m × 1.80 m (Figure 9b,c),
- for double-span elements B3C, two tripods of the optical system were used to cover the measurement areas of the support zone loaded with two forces and with one force, thus creating two measurement fields with dimensions of 0.30 m × 1.40 m each (Figure 9d). The surface of the measurement fields, shown in Figure 9, had to be adequately prepared before the test by applying a black paint pattern to enable continuous tracking of strain changes. Strain analyses and calculations were performed on the prepared measurement fields of the beams after the test ended.

### 2.5. Additional Tests

#### 2.5.1. Concrete Strength

**Figure 10:**
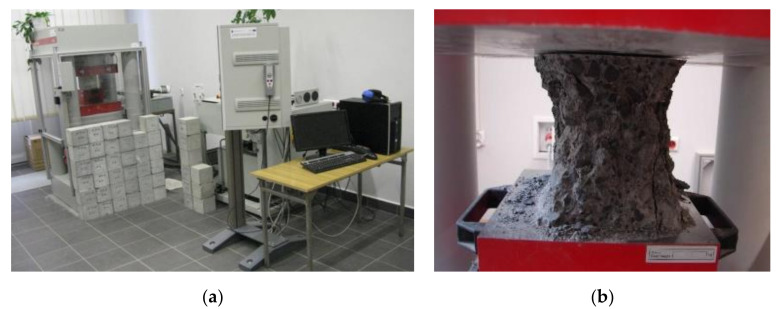
(**a**) Hydraulic press (**b**) An example image of concrete sample destruction.

The class of concrete and its strength characteristics were determined through axial compression tests (Figure 10) of concrete samples taken during beam manufacture. The elements were made in battery molds for two or four beams. Six cubic samples with dimensions 0.15 m × 0.15 m × 0.15 m and three cylinders with a diameter of 0.15 m and a height of 0.30 m were taken from each concrete mix batch. 

Table 4 summarizes the average values of concrete compressive strength f_cmCUBE_ obtained from the tests of concrete cubes, and f_cm_—average values of cylindrical strength of concrete estimated from the tests on cubic samples. Other values given in Table 4 include the strengths calculated using the formulas given in Eurocode 2 [14]: the characteristic value of strength on cubic samples—f_ckCUBE_, the characteristic value of the strength of concrete on cylindrical samples—f_ck_ and the average value of axial tensile strength f_ctm_.

**Figure 11:**
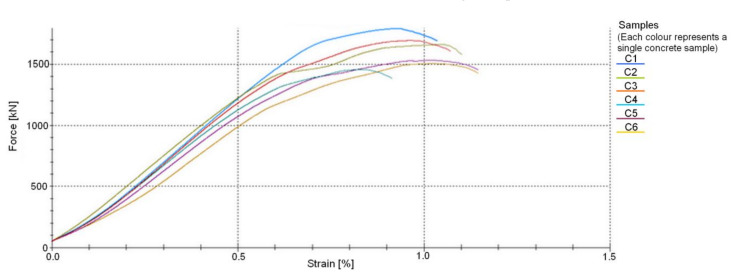
Force–strain relationships for concrete in beams B3C.

Figure 11 shows an example of a force–strain diagram of six concrete samples for one series of concrete beams B3C obtained using testXpert software. 

#### 2.5.2. Steel Strength

**Figure 12:**
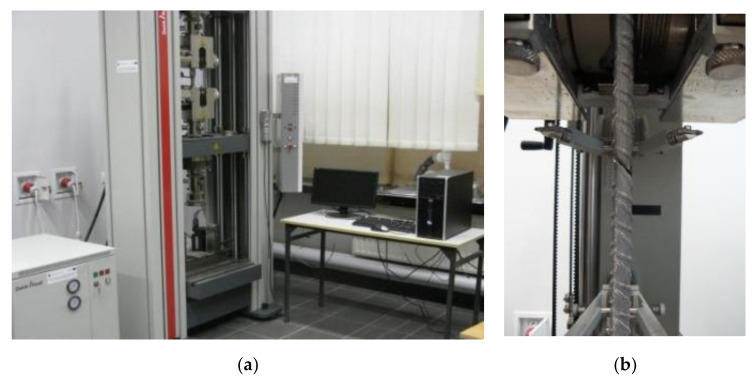
(**a**) Testing machine (**b**) An example image of the destruction of a steel sample.

The strength of the steel reinforcing bars used in the beams was determined through the axial tensile test of shear and longitudinal reinforcement samples (Figure 12). The reinforcing bar samples were taken during the fabrication of the elements and for control during the structural survey (Figure 2).

Table 5 shows the results of the average yield stress—tensile strength f_m_ of the reinforcing bars used as the longitudinal and shear reinforcement in individual beams. The table also shows the automatically obtained results of the elasticity modulus—E_s_ and the calculated characteristic values of the yield strength of the longitudinal reinforcement—f_yk_ and shear reinforcement f_ywk_.

**Figure 13:**
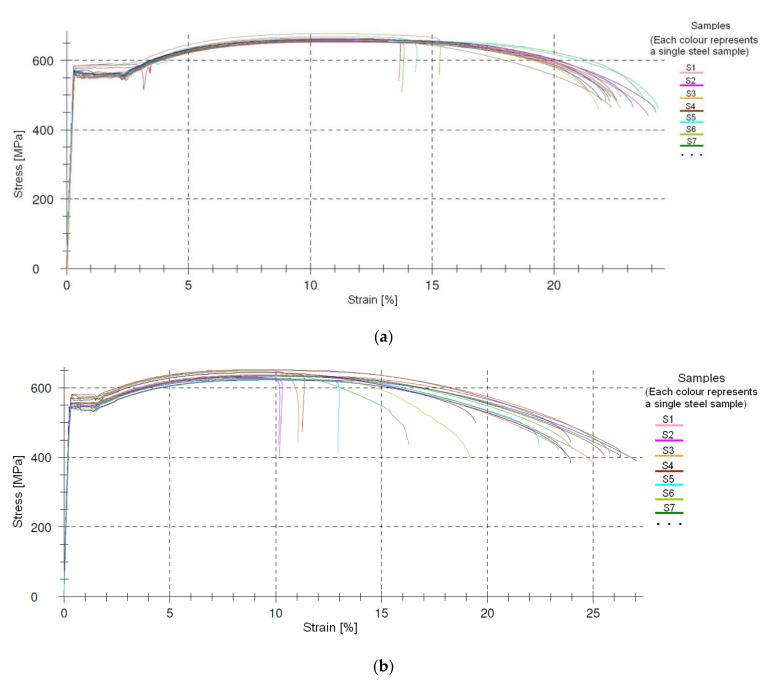
Stress–strain curve for bars (**a**) ϕ12, (**b**) ϕ14.

Due to the high repeatability of the results obtained for a given diameter and between diameters, the same steel parameters were adopted (Figure 13). The strength of the steel bars was estimated as the average value of all (about 100) bar axial tensile test results. 

## 3. Test Results and Analysis

### 3.1. Results of Experimental Research

**Figure 14:**
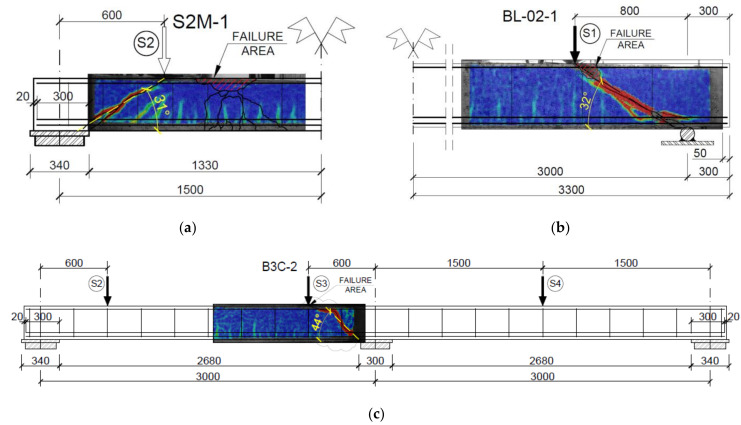
Examples of strain maps from beams (**a**) beam S2M-1, (**b**) beam BL-02-(M), (**c**) support zone surface of beam B3C-2.

**Figure 15:**
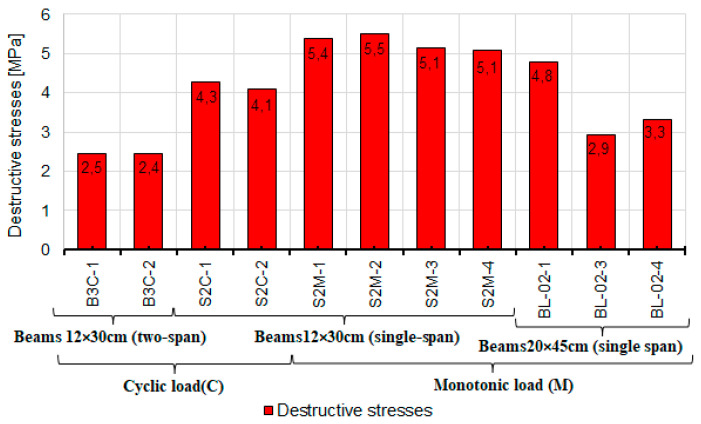
A diagram of shear stresses at failure of the tested elements.

Analysis of the shear capacity of reinforced concrete beams was primarily focused on the obtained load levels at which the element failed. Based on these levels, the values of the cross-sectional forces were determined, i.e., the shear force V and the bending moment M, given in Table 6. The DIC system (ARAMIS) helped determine the angle of inclination θ of the diagonal compression struts at the moment corresponding to the force destructive value. The angle was assumed to be equal to the angle of inclination of the diagonal cracks βr (Figure 14). The inclination angle was measured after the test from the strain maps of the prepared measurement fields on the side surface of the beams (Figure 9) recorded by the ARAMIS DIC system. Additionally, due to different geometries of the tested elements, the values of shear stresses at the neutral axis τ were determined for future comparisons based on shear forces (Figure 15) at failure, calculated from Formula (1).

(1) $$\tau =\frac{V}{b\cdot z}$$

where: V—shear force, *b*—width of the element, *z*—lever arm 

The calculated failure shear stresses in the beams under monotonic and cyclic loading are shown in Figure 15.

Examples of strain maps of the element’s side surface superimposed on reinforcement drawings are shown in Figure 14.

On the deformation maps corresponding to the measurement fields, which after completion of the test are assumed with the DIC (ARAMIS), the strains are marked using a color scale. The blue color marks the base area on which no strains are recorded yet. When they appear, the color of a given area becomes brighter, then changes to green and up to red, which indicates significant strains. In Figure 14, local accumulation of strains (brighter and then red) can be observed in the support zone of the beams, which for concrete (brittle material) is identified as cracks. In this way, the angle of inclination of the support zone diagonal cracks can be determined. 

### 3.2. Calculation Models

The development of built environments across the world has pushed forward R&D in concrete technology in the search for material saving opportunities and new methods for dimensioning reinforced concrete elements. Over the years of research, several shear capacity computational models have been developed to describe the behavior of the support zone under load. Formulated at the beginning of the 20th century by Mörsch [2], the classical theory assumed that concrete in the beam’s tension zone carries no tensile stresses at any cross-section, including between cracks. Hence, the principal stresses depend only on the shear stresses. This model—called the Mörsch truss—has survived for decades, and after various modifications was incorporated in different codes of practice, such as Polish standards [15,16], European standards [14,17], including ModelCode 2010 (MC2010) [3], and US standards [4,18]. The truss model has been greatly modified over the years, often significantly altering the classical Mörsch approach. New approaches were proposed by A. Ahmad and ALL Baker [19,20], F. Leonhardt and R. Walther [2,21], H. Kupfer and W. Dilger [22,23], H. Rüsch [24,25] or A. Lipski [26]. These studies, however, remained in the sphere of theoretical considerations and, mainly due to their complexity, only some of them found their application in engineering practice. 

Another approach to shear capacity of reinforced concrete elements is the ultimate failure state theory. It assumes that at the time of failure, the diagonal crack separates the beam into two parts connected by concrete in the non-cracked compression zone and by the reinforcement cut by that diagonal crack in the cracked zone. In this approach, the ultimate limit state of the concrete compression zone in the area above the so-called major diagonal crack is controversial because the strain in this area of concrete is determined by the combined effect of the moment and shear force. The precursor of this approach was M. S. Boriszański, who in 1937 began his research on the shear strength of reinforced concrete beams under the supervision of prof. A. A. Gwozdiew. The results of their analyses were published in [27]. Their method was later modified by Gyengö, Visa, Bay, Walther [2] and Kani [2,28,29]. Like its predecessor, the Boriszański state limit of failure approach was used in standard regulations, including the Soviet NiTU 123-55 and Polish standards [30]. Proposed changes or modifications to the classical theory developed and published by many researchers were often controversial. A compendium of knowledge about shear in reinforced concrete elements, truss models and the limit state method, with the conclusions, recommendations and guidelines from the author, is contained in the works of Godycki [2,20].

Studies related to the behavior of the support zone in reinforced concrete elements are still carried out in many research centers with improved apparatus and computational capabilities. Results of these studies have extended the existing body of knowledge and to the development of new theories and methods, such as the Modified Compression Field Theory [31] used in MC2010 [3], the Simplified Modified Compression Field Method [32,33], or the Generalized Stress Field approach [34].

Other, less known shear design approaches found in the literature include the shear zone model [35,36], dimensioning methods such as semi-probabilistic (e.g., Monte Carlo method) [37,38], finite element [39,40,41] or neural networks methods [42,43].

Due to the multitude of theoretical models for the support zone of reinforced concrete elements, only those that were or are the basis for standard regulations used in engineering practice were used for this analysis, with the concrete contribution in the shear load capacity of beams taken into account. These were primarily the models based on the ultimate state of failure (concrete contribution) [30], a truss model that does not include the contribution of concrete [16] also used in European standards [14,17], and the modified compression field method [32] accounting for the aggregate interlock [3,44,45,46].

Additionally, the analysis included models whose assumptions have been used in the provisions of American standards of 2014 and 2019 [4,18] and the calculation models [47], Caldera, Mari, Bairan, Oller And Ribas Method [48,49,50,51], Bentz and Collins [31,52], Reineck, [53], Park and Choi [54,55,56], Forsch, Yu, Cusatis and Bazant [57,58,59], Li, Hsu And Hwang [60,61,62,63,64], that led to the changes introduced in the US standard [4] of 2019 [65,66]. This choice was also dictated by the US approach to estimating the total shear bearing capacity of RC beams. It differs slightly from the European requirements. As a result, the calculations of the theoretical load capacity of the tested reinforced concrete beams were made on the basis of 21 models used in the European and US specifications.

### 3.3. Selection of Models for Calculation

A comparative analysis of the shear capacity results obtained from the tests and from the calculations was initially planned. The following calculation models were considered:

- Boriszański failure model [2,30],
- Mӧrsch truss model acc. to Kupfer–Rüsch [67] (PN-B-03264:2002) [16],
- Mӧrsch truss model acc. to Kupfer–Rüsch (PN-EN-1992-1:2008) [14],
- Mӧrsch truss model acc. to Kupfer–Rüsch (DIN 1045-1:2008) [17],
- models acc. to Model Code 2010; approximations 1, 2 and 3, [3,68],
- models acc. to ACI-318-14; approaches 1, 2, 3, 4 (ACI-318-14) [18],
- model acc. to ACI-318-19; approaches 1, 2, 3 (ACI-318-19) [4],
- Clader, Mari, Bairan, Oller and Ribas model, [48],
- Bentz and Collins model—simplified and detailed method, [52],
- Reineck model [53],
- Hong-Gun Park, Kyoung-Kyu Choi model [54],
- Forsch, Yu, Cusatis and Bazant model [57],
- Li, Hsu and Hwang [60].

However, as this number of models makes the comparison very difficult, a preliminary selection was performed based on experimental results. The experimental shear capacity of all tested elements was compared with the capacities calculated using all the models. The comparison was divided into two stages:

- comparison of the experimental load capacity with the shear capacity of the concrete cross-section in the beam supporting zone,
- comparison of the experimental load capacity with the shear capacity of the beam support zone.

As a result of the comparison, models that did not meet the assumed criteria were excluded from further analysis. The adopted criteria were:

- obtaining the calculated shear capacity of concrete or the calculated shear capacity for a given element greater than the experimental capacity,
- no repeatability of the model in the already selected group to avoid duplicating the results obtained,
- adopting substantial simplifications in the model in order to simplify the design calculations at the expense of the accuracy of the results.

The results of the initial verification are summarized in Table 7 and Table 8. Table 7 is for the theoretical load-bearing capacity of concrete, while Table 8 presents the total shear capacity results.

The results shown in Table 7 and Table 8 were the basis for excluding the following models (exceeded shear capacity of the support zone):

- Mӧrsch truss model acc. to Kupfer–Rüsch (PN-B-03264:2002) [16],
- Reineck model [53],
- ACI-318-14 model approach 4 [18],
- Li, Hsu and Hwang [60].

In addition, due to the significant simplifications adopted in the model, the modified Mӧrsch truss model used in the Model Code 2010 approximation 1 was omitted during the design [3]. Significant similarities between the models affected the exclusion of the following models:

- the model used in DIN 1045-1:2008 [17],
- the model used in ACI-318-14 approaches 1 and 2 [18],
- the model used in ACI-318-19 approaches 2 and 3 [4].

As a deviation from the adopted criteria, two models were kept:

- Boriszański failure model (PN-B-03264: 1984)—as the only model of the computational approach based on the limit state of failure [1,30], and,
- the simplified modified compressed field theory model used in Model Code 2010 approximation No. 3 [3]—as it is the only approach adopted in Model Code 2010, in which the total shear capacity of the support zone accounts for the resistance of cracked concrete expressed by aggregate interlock.

After the verification and consideration of individual solutions, the following models were finally adopted for further analysis:

- Boriszański model of failure (BOR) [2,30],
- Mӧrsch truss model acc. to Kupfer–Rüsch (PN-EN-1992-1-1:2008), (M-KR) [17],
- Model Code 2010, approximations 2 and 3, (MC2010 AP2 and AP3) [3],
- ACI-318-14 approach 3, (ACI-318-14 W3) [18],
- ACI-318-19 approach 1, (ACI-318-19 W1) [4],
- Bentz and Collins—detailed method, (BC-DM) [52],
- Cladera, Mari, Bairan, Oller and Ribas model, (CMBOR) [48],
- Forsch, Yu, Cusatis and Bazant model, (FYCB) [57],
- Hong-Gun Park, Kyoung-Kyu Choi model, (PC) [54].

Symbols that are used in the presented later graphs for a given model are in round brackets.

### 3.4. Analysis of the Results

The comparative analysis of the shear capacity of reinforced concrete beams determined based on selected calculation models and the capacity obtained from experimental tests was performed taking into account:

- the compliance of the shear capacity values obtained from the tests with those calculated according to the selected models,
- the effect of adopting the diagonal compression strut inclination angle θ,
- the contribution of concrete in shear resistance.

**Figure 16:**
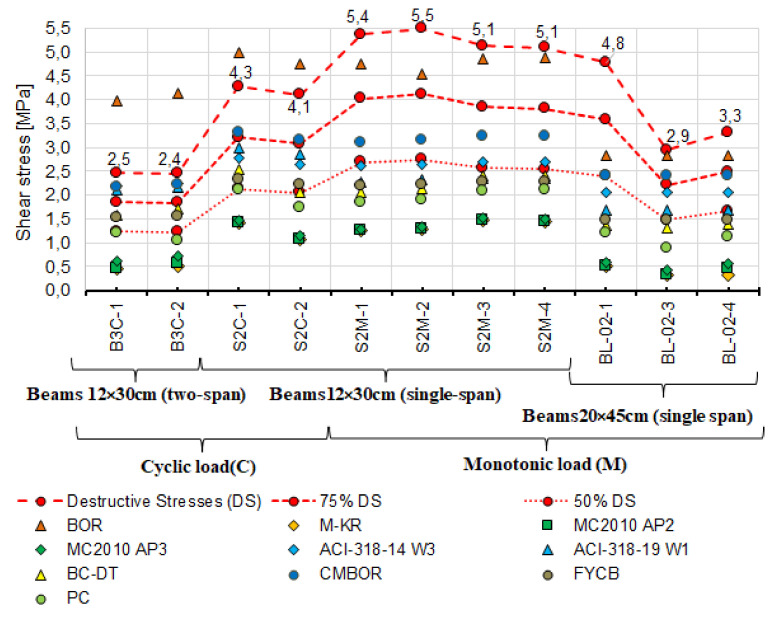
Comparison of destructive shear stresses obtained from the tests and calculated based on the real value of diagonal compression strut inclination angle θ_real_.

First, the experimental values and the calculated shear capacity were compared with each other. These values were determined by adopting the inclination angle of diagonal compression struts from the tests. The angle θ was adopted based on the inclination of the diagonal cracks βr (Figure 16). The values of angles θ from the tests are compiled in Table 6, and the calculated values of shear capacity are summarised in Table 9. Table 4 and Table 5 show experimentally determined concrete and steel strengths used in the calculations. 

It can be seen that shear capacity values calculated according to the failure state model (BOR) [2,30] exceed those obtained from the tests. The shear capacity is exceeded in four beams, 75% of the shear capacity in five beams, and 50% in three beams. The values of the shear capacity calculated from the remaining models are largely less than 75% of the experimental capacity. Furthermore, the capacities calculated using models based on the truss model, i.e.,:

- Mӧrsch truss by Kupfer–Rüsch (K.R.M.) (PN-EN-1992-1-1:2008), (M-KR) [14],
- Model Code 2010 approximations 2 and 3 (MC2010 AP2 and 3) [3],

are markedly lower than those from the tests. 

The lowest values of shear capacity obtained from the tests are for the two-span beams with variable low-cycle loads (B3C); it is more than two times less than that of simply supported beams with the same cross-section under monotonic loading (S2M). Despite the lower shear capacity of the B3C beams obtained in the tests, the calculated values from the selected models do not exceed this capacity. To a lesser extent, but analogous relationship occurs between the experimental and calculated shear values for single-span S2C beams under cyclic loading. For most of the models, the adopted value of θ has a significant impact on the calculated capacity of the reinforced concrete beams near the support zone. For this reason, the measured and estimated angles θ were additionally compared. The following models proposed four methods of estimating the angle: Model Code 2010 approximation 2 and 3 [3], the Bentz and Collins model [52] and the Hong-Gun, Kyoung-Kyu Choi model [54].

**Figure 17:**
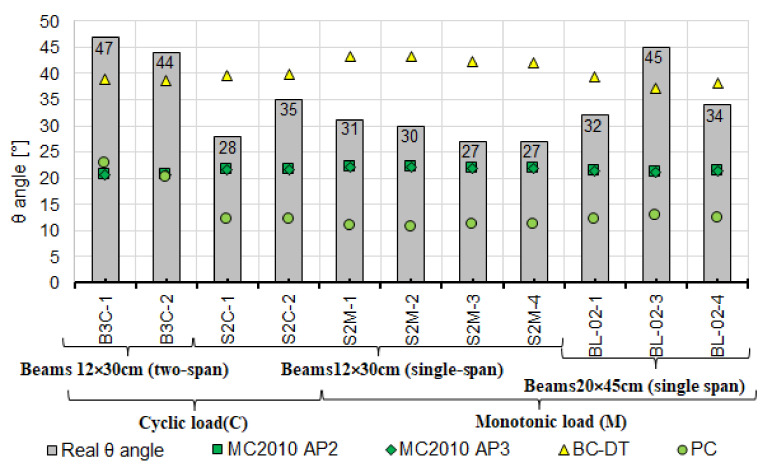
Comparison of the experimental and estimated values of angle θ.

The bar chart in Figure 17 compares the estimated and experimental values of angle θ. Bars represent angle θ corresponding to the diagonal crack’s inclination; the values estimated based on the models are shown using points varied in color and shape.

The greatest, more than two-fold difference between the measured and the estimated angles is observed for two-span beams under cyclic loading (B3C).

**Figure 18:**
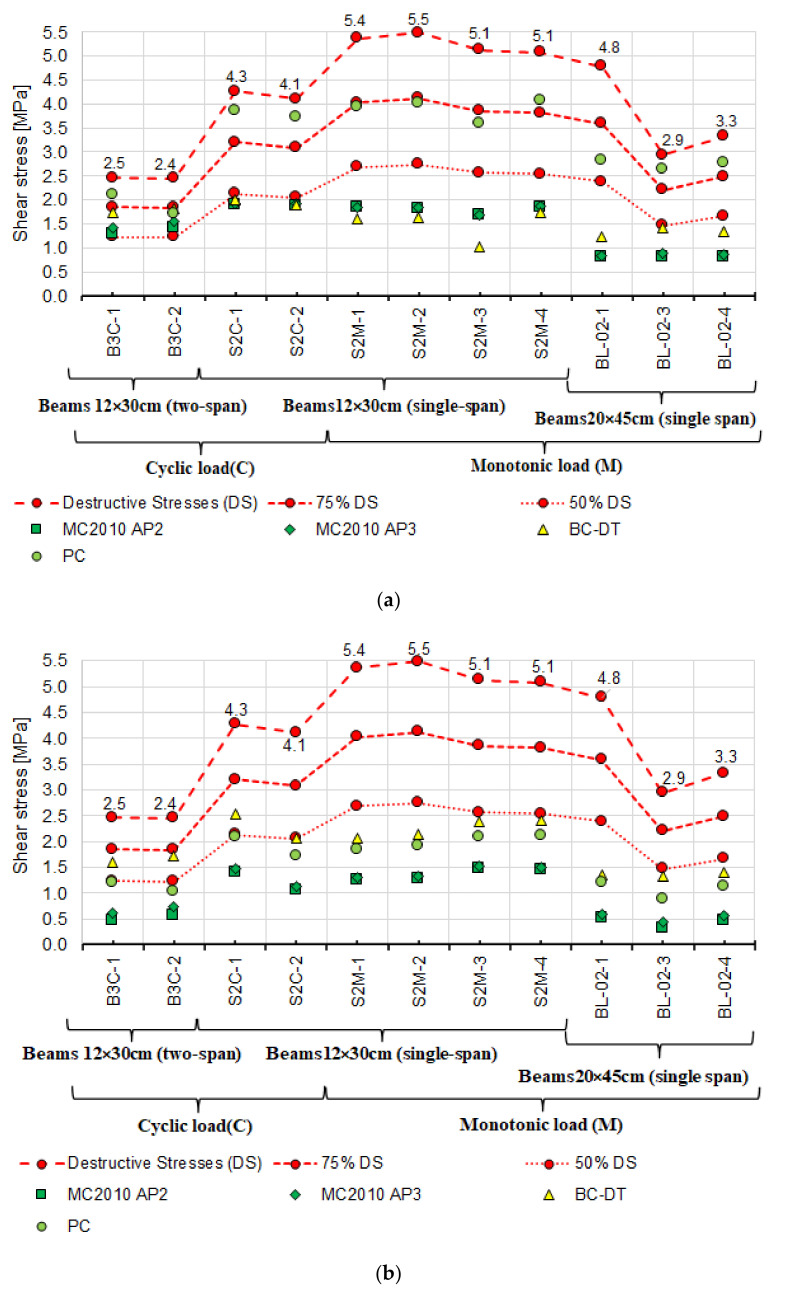
Comparison diagram of destructive shear stresses, experimental and calculated with (**a**) theoretical value of the θ_teo_ angle (**b**) real value of the θ_real_ angle.

Figure 18a illustrates the effect of adopting the estimated angle θ, corresponding to the angle of inclination of the concrete compression struts. It was developed analogously to Figure 16 but shows the calculated shear capacity only for those models where θ estimation is included. This applies to models:

- used in Model Code 2010, approximation 2 and 3 [3],
- Hong-Gun Park, Kyoung-Kyu Choi model [54],
- Bentz and Collins model—detailed method [52].

For a more precise illustration of the differences, the diagram in Figure 18b shows the shear capacity values calculated based on the angle θ measured in the tests, as in Figure 16. The calculations were narrowed down to the procedures including angle θ estimation methods. In the context of the differences seen in Figure 17, the comparison of the values in Figure 18 a and b reveals significant changes in shear capacity calculated based on the measured and estimated angles. Compared to shear capacity calculated using the measured θ, the largest difference is observed when the shear capacity is calculated according to the Hong-Gun Park model, Kyoung-Kyu Choi (PC) [54]. At the estimated angle θ, shear capacity values are higher than when the measured θ from experiments is adopted. For eight beams, the shear capacity values exceed 75% of the experimental shear capacity at almost twice as low values of the estimated angle θ, compared to the experimental θ, Figure 17.

Analysis of the results of the comparison between the experimental stresses and those calculated with the estimated angle θ (Figure 18a,b) shows reduced differences between the experimental shear capacity and calculated using the measured angle θ. Compared to the experimental shear capacity, the calculated values are still much lower. Only the capacities calculated using the Hong-Gun Park, Kyoung-Kyu Choi (PC) [54] model are greater than 75% of the experimental value. The same relation applies to the capacities calculated according to the model of Clader, Mari, Bairan, Oller and Ribas (C.M.B.O.R.) [48] (Figure 16), which is θ-independent. Therefore, it can be inferred that the values of θ adopted in calculations tend to play the role of a calibration factor. This is also confirmed by significant differences between the experimental and estimated angles θ, as shown in Figure 17.

Analysis of shear capacity of reinforced concrete beams should also account for the static scheme because statically indeterminate structural elements under variable loads dominate in building structures. Therefore, the beams loaded in a low-cycle manner were selected and subjected to additional extended analysis. After rejecting the models according to which the shear stresses are much lower and exceed (Figure 14) the experimental capacities, the analysis was performed using the following models:

- model used in ACI-318-14 approach 3 (ACI-318-14 W3) [18],
- model used in ACI-318-19 approach 1 (ACI-319-14 W1) [4],
- Bentz and Collins model—detailed method (B.C.-DT) [52],
- Cladera, Mari, Bairan, Oller and Ribas model (CMBOR) [48],
- Forsch, Yu, Cusatis and Bazant model (FYCB.) [57].

**Figure 19:**
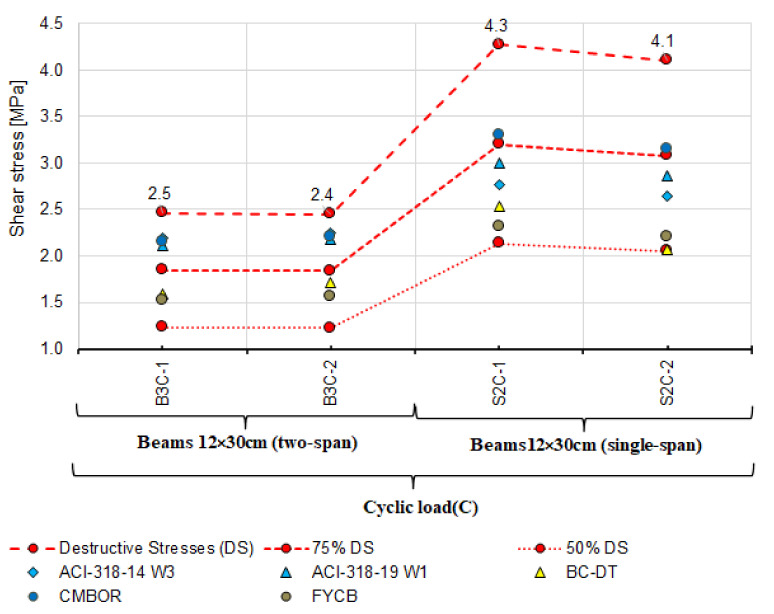
Comparison of experimental and theoretical shear stresses calculated based on the measured angle θ_real_ for beams loaded cyclically.

Figure 19 compares the results of experimental shear stresses based on the destructive loads and those calculated using the models above and the angle θ measured for beams loaded in a cyclic manner.

**Figure 20:**
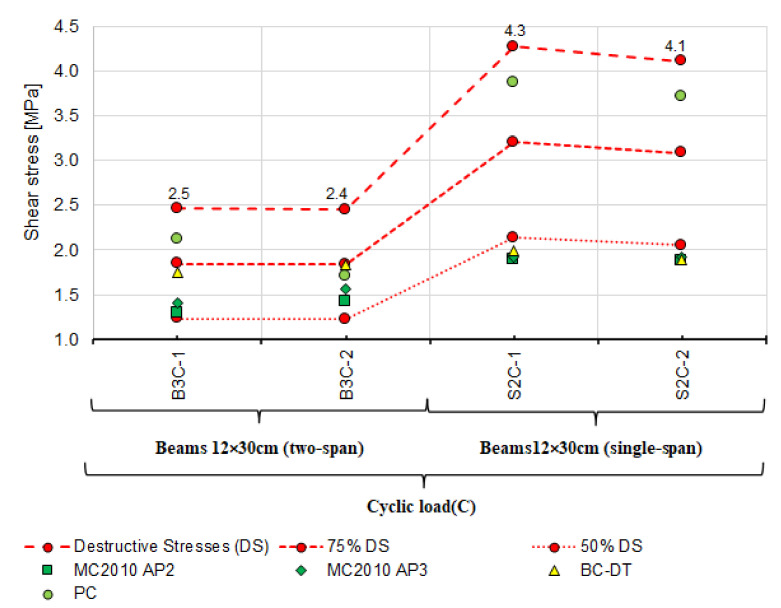
Comparison of experimental and theoretical shear stresses calculated based on the theoretical values of the angle θ_teo_ for beams loaded cyclically.

A similar comparison in terms of the θ effect on shear stresses is shown in Figure 20. The shear stresses represented by different point shapes were calculated with the estimated θ angle taken into account. The models used for the comparison are those that provide the method of estimating the angle θ. Shear stresses calculated using both the measured and the estimated angle θ are lower than those obtained from the tests. For statically determinate beams, practically all values of shear capacity are between 75% and 50% of the destructive shear stress, while for statically indeterminate beams, the shear stresses calculated based on the ACI-318-14 W3 [18], ACI-318-19 W1 [4] models and CMBOR [48] are close to the experimental results. It thus follows that for statically indeterminate beams, the models listed above provide the best agreement between the experimental and calculated shear capacity values.

Considering that some models include the concrete contribution term in shear capacity determination and some ignore its effect, the further analysis focused on the concrete contribution as provided by selected models. It is important because, depending on the calculation model, the values of the concrete shear capacity vary significantly. The models in which concrete contribution after cracking is included in shear capacity are:

- Boriszański model [2,30],
- Model Code 2010 approximation 3 [3],
- Hong-Gun Park, Kyoung-Kyu Choi [54],
- Bentz and Collins—simplified method [52],
- Cladera, Mari, Bairan, Oller and Ribas [48],
- Forsch, Yu, Cusatis and Bazant [57].

As the estimated concrete shear capacity is exceeded in:

- Mӧrsch truss model acc. to PN-EN-1992-1-1:2008 [14],
- Model Code 2010 approximation 2 [3],

the shear reinforcement is assumed to carry all shear stresses. 

It is worth noting that among the models showing the greatest correspondence between the calculated and experimental shear capacity, there are those in which both shear reinforcement contribution and concrete contribution are included. It seems that the greatest differences between the calculated and experimental shear capacity are obtained using the truss-based models (Figure 16 and Figure 18). Shear capacity calculated according to these models, after exceeding the formula-specified value (concrete shear capacity) results only from the capacity of shear reinforcement bars. This approach changed slightly in MC2010 AP3 [3] which added concrete contribution but only due to “aggregate interlocking” in the diagonal crack. This effect is computationally negligible, as evidenced by the results shown in Figure 16 and Figure 18. It is therefore justified to continue the considerations over determining the force that the cracked support zone concrete can carry and analyze the approaches in which, in addition to the shear reinforcement, the concrete contribution is taken into account to a greater extent [2,3,4,18,30,48,52,54,57]. For this purpose, Table 10 lists the shear capacities of RC beam support zone concrete accounted for in shear capacity values according to the models that include this capacity.

**Figure 21:**
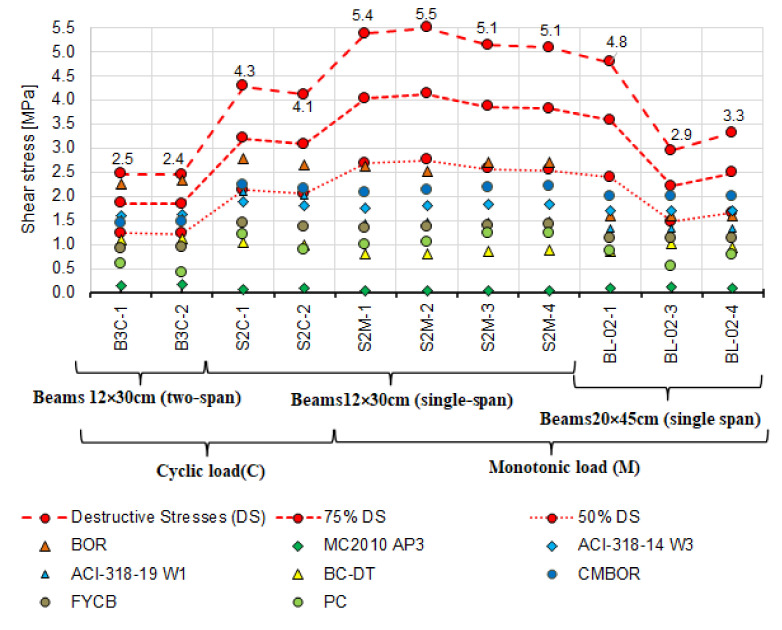
Destructive shear stresses and calculated concrete shear resistance.

**Figure 22:**
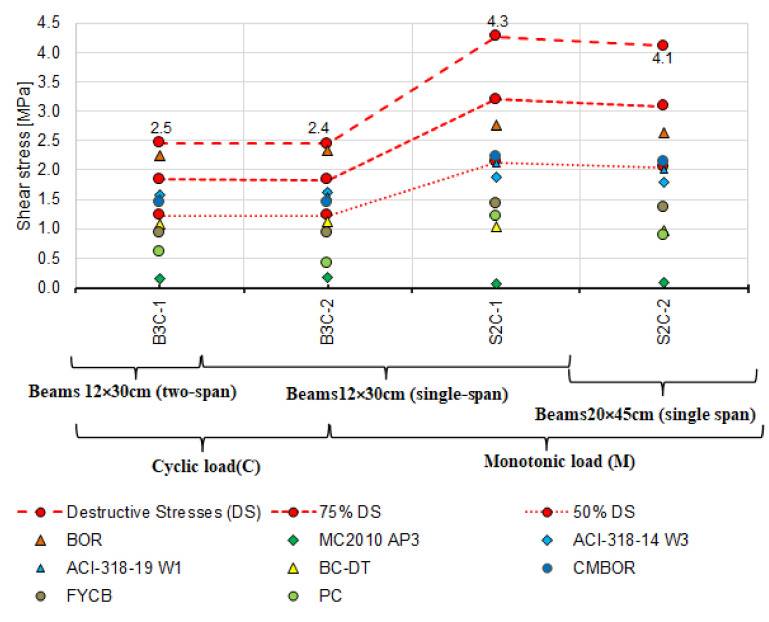
Destructive shear stresses and calculated concrete shear capacity for cyclically loaded elements.

Figure 21 and Figure 22 show the calculated concrete contribution relative to the shear capacity obtained based on destructive forces in the case of 11 reinforced concrete beams (Figure 21) and only cyclically loaded elements (Figure 22). The shear resistance of concrete in individual beams was marked with different point shapes representing different models. The destructive shear stresses are marked with red points connected by dashed lines. For comparative purposes, points connected by dashed lines corresponding to 75% of the experimental shear capacity are added.

From the figures above, it follows that the concrete contribution calculated with the procedures proposed in ACI-318-14 W3 [18], ACI-318-19 W1 [4], C.M.B.O.R. [48] in the experimental shear capacity exceeds 50%, particularly in the case of the statically indeterminate beams B3C loaded cyclically. It is only several percent when MC2010 AP3 [3] is used.

## 4. Discussion

Developments in concrete and steel technology have enhanced the strength and ductility of these materials, Figure 11 and Figure 13, generating a need for finding new dimensioning methods for reinforced concrete elements. The methods have to be based on scientifically warranted theoretical assumptions to account for higher strengths and ductility. This especially applies to the supporting zone of RC beams, where a complex state of stress occurs. This state of stress is additionally intensified by two phases, non-cracked and cracked, a reinforced concrete element experiences under service conditions. The resulting difficulties in describing the behavior of the support zones, most often referred to as shear, force the simplifications that must be verified experimentally. As the computational models proposed [2,19,20,21,22,23,24,25,26,27,28,29,30,31,32,33,34,35,36,37,38,39,40,41,42,43,44,45,46,47,48,49,50,51,52,53,54,55,56,57,58,59,60,61,62,63,64] fail to fully describe the real shear strength of reinforced concrete elements, the search for uniform models to formulate the shear design provisions is being continued. The models for incorporation in standard requirements are selected from among those that ensure the most reliable results for the adequate level of structural reliability. Therefore, it is important to experimentally verify previously and currently used models when developing the calculation model of the behavior of the supporting zone. Such a comparative analysis of the experimental to calculated values of shear capacity was performed. The elements under analysis differed in the static scheme, cross-sectional dimensions, and the loading mode and program. The analysis used computational models based on a series of modifications of the classic theory developed by Mörsch [2], the so-called truss model [3,14,15,16,17]. These included the ultimate state of failure model by Boriszański [2,30], the modified compressed field theory [3,31], the simplified modified compressed field theory [33], and the generalized stress field approach [30]. The verification was based on the test results of 11 shear-damaged reinforced concrete beams. The DIC system (ARAMIS) used in the study allowed non-contact and continuous tracking of the formation and development of diagonal cracks and the measurement of the angle of their inclination. 

The principal findings from the performed laboratory tests and comparative analyses are as follows:

- the loading program, static scheme, and the dimensions of the beam cross-section affect the experimental shear capacity of the reinforced concrete beam support zone,
- as demonstrated, statically indeterminate elements show lower shear capacity than statically determinate elements under cyclic and monotonic loads,
- based on the results in Figure 16, Figure 18, Figure 19 and Figure 20, verification tests of the calculation model should be carried out on statically indeterminate beams under cyclic loads,
- shear capacity estimated from the model based on the state of failure according to Boriszański (BOR) [2,30] exceeded the capacity obtained experimentally for four beams, which means that this model will not ensure the adequate level of structural reliability,
- values of shear capacity calculated from the modified truss model largely depend on the angle θ (the angle of inclination of a diagonal strut) and are much lower than the values obtained experimentally,
- the measured angle of the diagonal crack varies from the value calculated from the adopted models, in which the estimation method is given—Figure 17 (Model Code 2010 approximation 2 and 3 [3], the Bentz and Collins model [52] and the Hong-Gun Park model, Kyoung-Kyu Choi) [54,55,56],
- the ctg θ value used in some models does not result from the inclination angle θ of the compressed concrete diagonals and can be used as an effective parameter for adjusting the shear design values to fit the experimental results (e.g., Model Code 2010 approximations 2 and 3 [3], Bentz and Collins [52] and Hong-Gun Park, Kyoung-Kyu Choi model) [54,55,56],
- the highest agreement between the experimental and calculated shear capacity values was obtained from the models that do not depend on the angle θ (ACI-318-14 approach 3 [18], ACI-318-19 approach 1 [4], Cladera, Mari, Bairan, Oller and Ribas (CMBOR) [48]),
- the shear capacity of the RC beam support zone depends on the shear strength provided by the shear reinforcement and concrete (e.g., interlocking effect—MC2010 [3], dowel action—e.g., ACI [4,18]) during loading and after cracking. For this reason, it should be taken into account in shear capacity estimation.

## 5. Conclusions

The findings above lead to the following conclusions concerning the shear capacity of the support zone in reinforced concrete beams:

- verification of calculation models, including numerical models, should be based on test results for statically indeterminate beams under cyclic loading;
- the highest agreement between the experimental and calculated shear capacity was obtained from the models that are independent of angle θ;
- the value of ctg θ can serve as a parameter adjusting calculated shear capacity to real values;
- the support zone capacity calculations for the entire loading process and after cracking should take into account the capacities of both transverse reinforcement and concrete.

The comparative analysis conclusions were based on the tests of beams made of the same concrete class and steel type. The concrete had higher strength than the designed grade C40/50, and the ultimate strains exceeded 3.5 ‰ (Figure 12). The steel was characterized by very high ductility (Figure 13). Since the shear resistance is largely dependent on the properties of these materials, further research is required. It would be then possible to develop a database of research results allowing the verification of existing and newly developed models. Statistical analysis would ensure an adequate level of safety and reliability of structures.

The planned program of further shear tests of beams includes tests to supplement the material gap, i.e., tests of cyclically loaded double-span beams made of concrete class C30/37 and reinforcing steel class B, as well as high-strength concretes of approx. 90 MPa. Considering the importance of the fire resistance of reinforced concrete structures, tests should also be carried out for beam behavior at high temperatures, which has a significant impact on the adhesion of steel to concrete [69]. However, to do this, a broad testing program for members [70] at high temperatures is required along with adequate laboratory equipment. Future research directions include the development of a numerical model for the behavior of the support zone of reinforced concrete beams under load and its verification based on test results, as well as testing the shear capacity of reinforced concrete beams strengthened with composites and beams with non-metallic reinforcement.

## Figures and Tables

**Table 1:** Beam reinforcement.

Symbol	Dimension [m]		Longitudinal Reinforcement [mm]		Stirrups [mm/m]	Number of Elements [pcs]	Static Scheme
	Length	Cross-Section	Top	Bottom			
S2M-1 to 4	3.30	0.12 × 0.30	2ϕ8	3ϕ12 + 2ϕ14	ϕ4.5/0.20	4	Single-span
S2C-1,2	3.30	0.12 × 0.30	2ϕ8	3ϕ12 + 2ϕ14	ϕ4.5/0.20	2	Single-span
B3C-1,2	6.30	0.12 × 0.30	2ϕ12	3ϕ12 + 2ϕ14	ϕ4.5/0.30	2	Two-span
BL-02-M-1,3,4	6.60	0.20 × 0.45	2ϕ10	4ϕ20 + 2ϕ20	ϕ6.0/0.50	3	Single-span

**Table 2:** Summary of beam areas and longitudinal and shear reinforcement ratio.

Beams	Bottom Bars A_s1_ [cm^2^]	Average Bottom Reinforcement Ratio ρ_As1_	Top Bars A_s2_ [cm^2^]	Shear Reinforcement A_sw_ [cm^2^]	Shear Reinforcement Ratio ρ_w_
S2M-1 to 4	6.47	0.0212	1.01	0.32	0.0013
S2C-1,2	6.47	0.0212	1.01	0.32	0.0013
B3C-1,2	4.52	0.0148	2.26	0.32	0.0009
BL-02-M-1,3,4	18.85	0.0235	1.57	0.32	0.0009

**Table 3:** Levels and frequencies of individual beams loading.

Beam	Range I [kN]	Frequency I f [Hz]	Range II [kN]	Frequency II f [Hz]	Range III [kN]	Frequency III f [Hz]
S2C-1,2	5–30	0.5	5–70	0.5	5–110	0.3
B3C-1,2	15–25	0.25	15–35	0.12	15–55	0.06

**Table 4:** Basic parameters of concrete.

Beams		f_cm_^CUBE^ [MPa]	f_cm_ [MPa]	f_ck_^CUBE^ [MPa]	f_ck_ [MPa]	f_ctm_ [MPa]
Single-span beams 0.12 m × 0.30 m × 3.30 m	S2M-1,2	71.9	57.5	63.9	49.5	4.0
	S2M-3,4	74.1	59.3	66.1	51.3	4.1
	S2C-1,2	74.1	59.3	66.1	51.3	4.1
Two-span beams 0.12 m × 0.30 m × 6.30 m	B3C-1,2	71.2	57.0	63.2	49.0	4.0
Single-span beams 0.20 m × 0.45 m × 6.60 m	BL-02-1	65.2	52.1	57.2	44.1	3.7
	BL-02-3					
	BL-02-4					

**Table 5:** Basic parameters of steel.

Beams		f_m_ [MPa]	f_yk_ [MPa]	f_ywk_ [MPa]	E_s_ [GPa]
Single-span beams 0.12 m × 0.30 m × 3.30 m	S2M-1,2,3,4	646.0	562.5	559.6	199.7
	S2C-1,2				
Two-span beams 0.12 m × 0.30 m × 6.30 m	B3C-1				
	B3C-2				
Single-span beams 0.20 m × 0.45 m × 6.60 m	BL-02M-1				
	BL-02M-3				
	BL-02M-4				

**Table 6:** A list of the destructive loads of reinforced concrete beams with the corresponding cross-sectional forces and the angles of inclination of compression struts.

Beams	Angle θ [deg.]	Destructive Load				Shear Force and Bending Moment at the Destructive Load		Shear Stress τ [MPa]
		S1 [kN]	S2 [kN]	S3 [kN]	S4 [kN]	V [kN]	M [kNm]	
S2M-1	31	-	150.6	149.8	-	151.3	90.9	5.4
S2M-2	30	-	147.5	152.2	-	152.1	91.4	5.5
S2M-3	27	-	134.7	134.7	-	135.6	81.5	5.1
S2M-4	27	-	134.7	133.2	-	134.3	80.8	5.1
S2C-1	28	-	109.8	109.7	-	110.5	66.5	4.3
S2C-2	35	-	109.7	109.8	-	110.6	66.5	4.1
B3C-1	47	-	54.8	54.8	15.8	64.0	15.5	2.5
B3C-2	44	-	54.9	54.8	15.3	64.1	15.4	2.4
BL-02-1	32	402.0	-	-	-	353.4	283.4	4.8
BL-02-3	45	261.1	-		-	217.5	240.6	2.9
BL-02-4	34	295.3	-		-	245.5	271.3	3.3

**Table 7:** A summary of the concrete load-bearing capacity of the reinforced concrete beam support zone according to selected calculation models with color marking of the models selected for further analysis.

Model	B3C-1	B3C-2	S2C-1	S2C-2	S2M-1	S2M-2	S2M-3	S2M-4	BL-02-1	BL-02-3	BL-02-4
Destructive Stresses (D.S.)	2.5	2.4	4.3	4.1	5.4	5.5	5.1	5.1	4.8	2.9	3.3
75% D.S.	1.8	1.8	3.2	3.1	4.0	4.1	3.8	3.8	3.6	2.2	2.5
50% D.S.	1.2	1.2	2.1	2.1	2.7	2.7	2.6	2.5	2.4	1.5	1.7
Mӧrsch truss analogy by Kupfer–Rüsch (PN-B-03264:2002) [16]	3.3	3.4	3.6	3.5	3.4	3.4	3.5	3.5	2.7	2.7	2.7
ACI-318-14 W4 [18]	2.6	2.7	2.6	2.5	2.5	2.5	2.6	2.6	2.3	2.3	2.3
Reineck [53]	2.7	2.8	2.8	2.6	2.6	2.6	2.7	2.7	2.3	2.3	2.3

**Table 8:** A summary of the total load-bearing capacity of the reinforced concrete beam support zone according to selected calculation models with color marking of the models selected for further analysis.

Model	B3C-1	B3C-2	S2C-1	S2C-2	S2M-1	S2M-2	S2M-3	S2M-4	BL-02-1	BL-02-3	BL-02-4
Destructive Stresses (D.S.)	2.5	2.4	4.3	4.1	5.4	5.5	5.1	5.1	4.8	2.9	3.3
75% D.S.	1.8	1.8	3.2	3.1	4.0	4.1	3.8	3.8	3.6	2.2	2.5
50% D.S.	1.2	1.2	2.1	2.1	2.7	2.7	2.6	2.5	2.4	1.5	1.7
Li, Hsu and Hwang [60]	2.4	2.5	2.8	2.6	2.6	2.7	2.7	2.7	2.1	1.7	1.7

**Table 9:** Shear capacity of reinforced concrete beams according to selected models, adopting the angle θ from the tests.

Model	B3C-1	B3C-2	S2C-1	S2C-2	S2M-1	S2M-2	S2M-3	S2M-4	BL-02-1	BL-02-3	BL-02-4
Destructive Stresses (D.S.)	2.5	2.4	4.3	4.1	5.4	5.5	5.1	5.1	4.8	2.9	3.3
75% D.S.	1.8	1.8	3.2	3.1	4.0	4.1	3.8	3.8	3.6	2.2	2.5
50% D.S.	1.2	1.2	2.1	2.1	2.7	2.7	2.6	2.5	2.4	1.5	1.7
Boriszański Failure Model (PN-B-03264:1984) [2,30]	4.0	4.1	5.0	4.8	4.7	4.5	4.9	4.9	2.8	2.8	2.8
Mӧrsch truss analogy by Kupfer–Rüsch (PN-EN-1992-1-1:2008) [14]	0.5	0.5	1.4	1.1	1.2	1.3	1.5	1.5	0.5	0.3	0.3
Generalized stress field approach (Model Code 2010 AP2) [3]	0.5	0.6	1.4	1.1	1.2	1.3	1.5	1.5	0.5	0.3	0.5
Simplified modified compression field theory (Model Code 2010 AP3) [3]	0.6	0.7	1.5	1.1	1.3	1.3	1.5	1.5	0.6	0.4	0.6
ACI-318-14 W3 [18]	2.2	2.2	2.8	2.6	2.6	2.7	2.7	2.7	2.1	2.1	2.1
ACI-318-19 W1 [4]	2.1	2.2	3.0	2.9	2.3	2.3	2.3	2.4	1.7	1.7	1.7
Bentz and Collins—detailed method [52]	1.7	1.7	2.6	2.0	2.3	2.4	2.6	2.6	1.5	1.3	1.4
Cladera, Mari, Bairan, Oller and Ribas [48]	2.2	2.2	3.3	3.2	3.1	3.1	3.2	3.2	2.4	2.4	2.4
Forsch, Yu, Cusatis and Bazant [57]	1.5	1.6	2.3	2.2	2.2	2.2	2.3	2.3	1.5	1.5	1.5
Hong-Gun Park, Kyoung-Kyu Choi [54]	1.2	1.0	2.1	1.7	1.8	1.9	2.1	2.1	1.2	0.9	1.1

**Table 10:** Shear capacity of reinforced concrete beams according to selected models, assuming the angle θ from the tests.

Model	B3C-1	B3C-2	S2C-1	S2C-2	S2M-1	S2M-2	S2M-3	S2M-4	BL-02-1	BL-02-3	BL-02-4
Destructive Stresses (D.S.)	2.5	2.4	4.3	4.1	5.4	5.5	5.1	5.1	4.8	2.9	3.3
75% D.S.	1.8	1.8	3.2	3.1	4.0	4.1	3.8	3.8	3.6	2.2	2.5
50% D.S.	1.2	1.2	2.1	2.1	2.7	2.7	2.6	2.5	2.4	1.5	1.7
Boriszański Failure Model [2,30]	2.3	2.3	2.8	2.6	2.6	2.5	2.7	2.7	1.6	1.6	1.6
Simplified modified compression field theory (* Model Code 2010 AP3) [3]	0.16	0.18	0.07	0.08	0.05	0.04	0.04	0.04	0.08	0.12	0.10
ACI-318-14 W3 [18]	1.6	1.6	1.9	1.8	1.8	1.8	1.8	1.8	1.7	1.7	1.7
ACI-318-19 W1 [4]	1.5	1.6	2.1	2.0	1.4	1.5	1.5	1.5	1.3	1.3	1.3
Bentz and Collins—detailed method [52]	1.1	1.1	1.1	0.9	1.0	1.1	1.1	1.1	1.0	1.0	1.0
Cladera, Mari, Bairan, Oller and Ribas [48]	1.4	1.5	2.2	2.1	2.1	2.1	2.2	2.2	2.0	2.0	2.0
Forsch, Yu, Cusatis and Bazant [57]	0.9	0.9	1.4	1.4	1.3	1.4	1.4	1.4	1.1	1.1	1.1
Hong-Gun Park, Kyoung-Kyu Choi [54]	0.6	0.4	1.2	0.9	1.0	1.0	1.2	1.2	0.8	0.5	0.8

## Data Availability

Data available in a publicly accessible repository.
